# Safety of Teacrine^®^, a Non-Habituating, Naturally-Occurring Purine Alkaloid Over Eight Weeks of Continuous Use

**DOI:** 10.1186/1550-2783-12-S1-P59

**Published:** 2015-11-12

**Authors:** Sara Hayward, Jacy Mullins, Stacie Urbina, Emily Santos, Katelyn Villa, Nicole Viana, Shelbey Perkins, Jennifer Ander, Alyssa Olivencia, Shannon McGorty, Colin Wilborn, Lem Taylor

**Affiliations:** 1 Department of Exercise & Sports Science, Human Performance Lab, University of Mary Hardin-Baylor, Belton, TX 76531, USA

## Background

Theacrine (1,3,7,9-tetramethyluric acid) is a purine alkaloid found in certain coffee (Coffea) species, fruits (Cupuacu [*Theobroma grandiflorum*]), and tea (*Camellia assamica, var. kucha*) that has anti-inflammatory, analgesic, and neuro-locomotor properties. Recent preliminary research has also reported increased feelings of energy, reduced fatigue, and strong effects on improving focus, concentration, motivation to exercise and libido.

## Purpose

To examine the safety and non-habituating effects of Teacrine^®^, a nature-identical, chemically equivalent bioactive version of theacrine.

## Methods

Sixty healthy men and women (mean ± SD age, height, weight: 22.55 ± 4.6 yr, 174.09 ± 12.4 cm, 77.47 ± 17.4 kg) were placed into one of three groups: placebo (P, n = 20), 200 mg Teacrine^® ^(T2, n = 19) or 300 mg Teacrine^® ^(T3, n = 21) and ingested their respective supplement once daily for eight weeks. Primary outcomes were fasting clinical safety markers (heart rate, blood pressure, lipid profiles, hematologic blood counts, biomarkers of liver/kidney/immune function) and energy, focus, concentration, anxiety, motivation to exercise, and POMS measured prior to daily dosing to ascertain potential tachyphylactic responses and discontinuation effects. Data were analyzed via ANOVA/ANCOVA and statistical significance was accepted at p ≤ 0.05.

## Results

All values for clinical safety markers fell within normal limits and no group × time interactions were noted. No habituation effects were noted as baseline values for energy, focus, concentration, anxiety, motivation to exercise, and POMS remained stable among groups. No serious adverse events were identified, and no differences in side effect profiles were detected between groups.

## Conclusion

These findings support the clinical safety and non-habituating neuro-energetic effects of Teacrine^® ^supplementation over eight weeks of daily use (up to 300 mg/day). Moreover, there was no evidence of a tachyphylactic response that is typical of neuroactive agents such as caffeine and other stimulants.

## Competing interests

This study was funded in part by a research grant from Compound Solutions, Inc (Carlsbad, CA).

